# Research progress on the E protein of porcine reproductive and respiratory syndrome virus

**DOI:** 10.3389/fmicb.2023.1139628

**Published:** 2023-05-15

**Authors:** Xiuqiao Chen, JingHua Pan, Liangzong Huang, Mengmeng Zhao

**Affiliations:** ^1^School of Life Science and Engineering, Foshan University, Foshan, China; ^2^Veterinary Teaching Hospital, Foshan University, Foshan, China

**Keywords:** PRRSV, E protein, ORF2b, ion channel, transmissible

## Abstract

Porcine reproductive and respiratory syndrome (PRRS) is an economically important disease impacting the global pig industry, and it is characterized by reproductive disorder in sows and respiratory disorder in pigs of all ages. The PRRSV E protein is a nonglycosylated structural protein encoded by the ORF2b gene. The E protein is not necessary for the assembly of virus particles, but deletion of the E protein leads to transmissible virus particles not being produced. To better understand the structure and function of the E protein, we reviewed its genetic and evolutionary analysis, characteristics, subcellular localization and topology, ion channel activity, cellular immune response, additional biological functions, interactions with host proteins, interactions with PRRSV proteins, roles in infection, pathogenicity, and drugs. Therefore, this review can provide a theoretical basis for gaining an in-depth understanding of the E protein of PRRSV-2.

## 1. Introduction

Porcine reproductive and respiratory syndrome (PRRS), a highly contagious infectious disease caused by porcine reproductive and respiratory syndrome virus (PRRSV), is characterized by reproductive disorder in sows and respiratory disorder in pigs of all ages and is commonly known as “blue ear disease” (Sun et al., 2011; Li et al., 2018). PRRS was first reported in America in 1987 (Keffaber, 1989). In a few short years, PRRSV quickly spread worldwide and became an economically important disease endangering the global pig industry. In 1996, Guo et al. isolated PRRSV for the first time in China, confirming the presence of PRRS in China (Guo et al., 1996).

PRRSV is an enveloped, single-stranded RNA virus belonging to *Arteriviridae* (Adams et al., 2016). According to the classification approved by the International Committee on Taxonomy of Viruses in 2017, PRRSV is divided into two species, namely, the European species (PRRSV-1, representative strain Lelystad) and the North American species (PRRSV-2, representative strain VR-2332; Shi et al., 2010; Adams et al., 2017; Ruedas-Torres et al., 2021; Walker et al., 2021). The shared nucleotide sequence similarity between the two species is 50–70% (Murtaugh et al., 1995; Kappes and Faaberg, 2015). The genome size of PRRSV is approximately 15.3 kb, with twelve open reading frames (ORFs) encoding eight structural proteins and 16 nonstructural proteins (Li et al., 2015). The eight structural proteins are GP2a, GP3, GP4, GP5, membrane (M) protein, envelope (E) protein, N protein and GP5a (Yuan et al., 2022). The PRRSV E protein is a nonglycosylated structural protein with a length of 70–73 aa encoded by the ORF2b gene. The E protein has a single cross-helical structure and ion channel activity and is the minor structural protein of virus particles (Wu et al., 2005; García Durán et al., 2016). The E protein is not required for the assembly of virus particles, but virus particles with E protein deletions are not contagious (Lee and Yoo, 2006). As a minor structural protein, the E protein plays an important role in the formation of infectious PRRSV particles.

## 2. Genetic evolution analysis of the E protein sequence

The PRRSV E protein sequence is highly conserved, especially the hydrophilic C-terminus. Yu et al. (2010) compared the E protein sequences of 235 PRRSV-2 isolates, and the amino acid sequence analysis and the E protein hydrophilic pattern map showed that the amino acids at most positions of the E protein were strictly conserved. Most positions prone to significant variability were occupied by residues with hydrophobic properties. Therefore, although the E protein sequence showed significant amino acid variability at several positions, the conservation of hydrophilic amino acid residues and hydrophobic amino acid residues at most positions revealed strong conservation of the E protein structure and organization in the PRRSV isolate.

**Figure 1:**
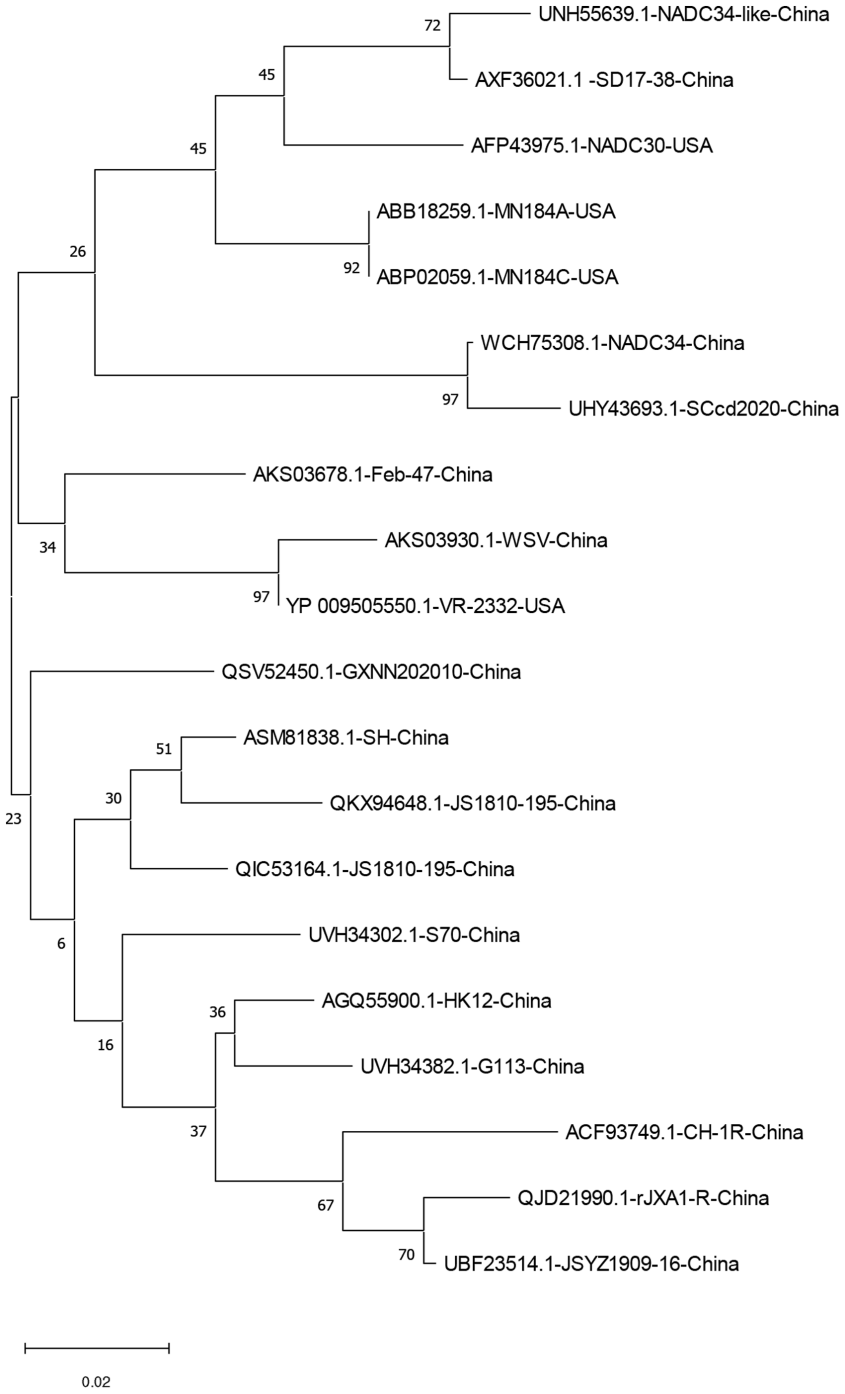
A neighbor-joining phylogenetic tree was constructed based on the E protein sequences of 20 PRRSV strains using MEGA software (ver. 11.0) with 1,000 bootstrap replicates.

The homology of twenty PRRSV E protein sequences was analyzed using the MegAlin program of DNASTAR Lasergene 7.1 software. The results revealed a homology range of 86 to 100% among the sequences. Twenty E protein sequences of PRRSV were downloaded from the NCBI database (Table 1), and a phylogenetic tree analysis was performed based on the E protein sequences using the MEGA neighbor-joining algorithm (NJ method; Figure 1). The phylogenetic tree results showed that the genetic distances among the 20 PRRSV E protein sequences were small. Multiple sequence alignment of the 20 PRRSV E proteins revealed 54 conserved sites and 19 variant sites in the PRRSV-2 E protein sequence. The C-terminus was highly conserved (amino acids 69–72) among these proteins (Table 2).

**Table 1:** Reference sequences of 20 PRRSV strains.

Serial number	Species	Accession number	Area	Poison strain
1	PRRSV-2	UNH55639.1	China	NADC34-like
2	PRRSV-2	WCH75308.1	China	NADC34
3	PRRSV-2	ABB18259.1	USA	MN184A
4	PRRSV-2	ABP02059.1	USA	MN184C
5	PRRSV-2	AFP43975.1	USA	NADC30
6	PRRSV-2	AGQ55900.1	China	HK12
7	PRRSV-2	ASM81838.1	China	SH
8	PRRSV-2	QIC53164.1	China	JS1810-195
9	PRRSV-2	UVH34302.1	China	S70
10	PRRSV-2	AKS03678.1	China	Feb-47
11	PRRSV-2	AKS03930.1	China	WSV
12	PRRSV-2	UVH34382.1	China	G113
13	PRRSV-2	QSV52450.1	China	GXNN202010
14	PRRSV-2	YP_009505550.1	USA	VR-2332
15	PRRSV-2	QJD21990.1	China	rJXA1-R
16	PRRSV-2	QKX94648.1	China	JSTZ1810-220
17	PRRSV-2	UBF23514.1	China	JSYZ1909-16
18	PRRSV-2	ACF93749.1	China	CH-1R
19	PRRSV-2	UHY43693.1	China	SCcd2020
20	PRRSV-2	AXF36021.1	China	SD17-38

**Table 2:** C-terminal conserved sequences of PRRSV-2.

	69	70	71	72
UNH55639.1-NADC34-like-China	L	Q	K	I
WCH75308.1-NADC34-China	.	.	.	.
ABB18259.1-MN184A-USA	.	.	.	.
ABP02059.1-MN184C-USA	.	.	.	.
AFP43975.1-NADC30-USA	.	.	.	.
AGQ55900.1-HK12-China	.	.	.	.
ASM81838.1-SH-China	.	.	.	.
QIC53164.1-JS1810-195-China	.	.	.	.
UVH34302.1-S70-China	.	.	.	.
AKS03678.1-Feb-47-China	.	.	.	.
AKS03930.1-WSV-China	.	.	.	.
UVH34382.1-G113-China	.	.	.	.
QSV52450.1-GXNN202010-China	.	.	.	.
YP 009505550.1-VR-2332-USA	.	.	.	.
QJD21990.1-rJXA1-R-China	.	.	.	.
QKX94648.1-JS1810-195-China	.	.	.	.
UBF23514.1-JSYZ1909-16-China	.	.	.	.
ACF93749.1-CH-1R-China	.	.	.	.
UHY43693.1-SCcd2020-China	.	.	.	.
AXF36021.1-SD17-38-China	.	.	.	.

## 3. Characteristics of the E protein

In 1999, a new 67 aa structural protein encoded by ORF2a was found in equine arteritis virus (EAV). The genome sequences of EAV and lactate dehydrogenase-elevating virus (LDV), PRRSV and simian haemorrhagic fever virus (SHFV), which belong to the same virus family, *Arteriviridae,* were analyzed. EAV ORF2a homologous genes were identified in LDV (ORF2a), PRRSV (ORF2b) and SHFV (ORF4a). To avoid confusion caused by differences in the numbering of the novel genes (ORF2a, ORF2b, and ORF4a) in the genomes of different arteriviruses, the newly discovered gene coding products were named the E proteins (Snijder et al., 1999). The E protein of PRRSV is a nonglycosylated protein (Li et al., 2010; Tian et al., 2012) that consists of a central hydrophobic structure (amino acids 14–54), a hydrophilic C-terminus containing a basic residue cluster and an N-terminus with a potential N-cardamom acylation site and a casein kinase II phosphorylation site (Liu et al., 2011; Zhang et al., 2012). The N-terminus of the E protein contains a conserved myristoylation motif (1MGxxxS6), myristoylation of which can promote viral growth (Wu et al., 2001; Du et al., 2010). The E protein has two cysteines, at positions 49 and 54, which are highly conserved in North American isolates, but the cysteine residues in the E protein are not necessary for the replication of the North American PRRSV species (Lee and Yoo, 2005).

## 4. Subcellular localization and topological structure of the E protein

Yu et al. cotransfected cells with the PRRSV E protein and organelle markers (Yu et al., 2010). The results of fluorescence confocal microscopy showed that endoplasmic reticulum markers (pDsRed2-ER) and Golgi markers (pEYFP-GOLGI) had the same staining pattern as the PRRSV E protein, which confirmed that the E protein was primarily distributed in the endoplasmic reticulum and Golgi complex. Yu et al. used a truncated E protein fused with EGFP and confirmed that the E protein contains three localization domains, located in the first 15 residues, residues 23–50 and residues 50–73, and the 15 N-terminal residues are the ER localization sequence of the E protein (Yu et al., 2010). Different buffers were used to treat the membrane portion, and the proteins with transmembrane and peripheral membrane interactions were distinguished. The results showed that the E protein did not bind to the periphery of the inner membrane cavity, which indicated that the E protein was a complete membrane protein embedded in the phospholipid bilayer, and mutation of the N-terminal acylation site and truncation of the conserved C-terminal region did not affect the membrane binding of the E protein. Topological analysis showed that the N-terminus of the E protein was oriented toward the cytoplasm and the C-terminus was oriented toward the endoplasmic cavity (Yu et al., 2010; Veit et al., 2014).

## 5. Ion channel activity of the E protein

The PRRSV E protein has ion channel activity, which is necessary for PRRSV infection. PRRSV infection requires an acidic environment (Kreutz and Ackermann, 1996; Tian et al., 2014). Therefore, MARC-145 cells infected with PRRSV were treated with ammonium chloride, chloroquine, amantadine and verapamil, and a chain-specific reverse transcription-polymerase chain reaction (RT–PCR) test was performed. Among these treatments, amantadine is a proton channel blocker, and verapamil is a calcium channel blocker. The results showed that MARC-145 cells infected with PRRSV treated with amantadine and verapamil did not exhibit virus production 1 day after infection, and the virus production titer reached only 5–8 × 10^1^ PFU/mL at 3 days after infection, which confirmed that the PRRSV E protein was an ion channel protein. Ion channel blockers effectively interfered with the shelling of PRRSV, inhibited the initiation of viral RNA synthesis, and affected the production of PRRSV (Lee and Yoo, 2006).Viroporins are usually small viral transmembrane membrane proteins with hydrophobic structures (Opella, 2015) and have a molecular weight of 6 kDa-12 kDa. Viroporins have a high α helical content and hydrophobicity and can form pores on the host cell membrane through oligomerization. The E protein is encoded by ORF2b of PRRSV and has a hydrophobic domain, making it a type of viroporin (Xu et al., 2019). It exhibits a characteristic change in membrane permeability and oligomerization (Pinto et al., 1992). The permeability of hygromycin B was measured based on the ability to penetrate the permeable membrane and cause strong inhibition of intracellular protein synthesis. The results showed that hygromycin B entered cells expressing the E protein and blocked intracellular protein synthesis, which confirmed that the PRRSV E protein enhanced the membrane permeability of the cells.

## 6. The E protein regulates the cellular immune response

Tetherin, a type II transmembrane protein induced by IFN-I, has a unique topological structure that can connect virus particles to the surface of the cell membrane during virus budding and limit the effective release of the virus, thus exerting broad-spectrum antiviral activity (Yi et al., 2014). pCMV-E-HA, pCMV-IFITM1 and pCMV-Tetherin-Myc were cotransfected into human embryonic kidney (HEK) 293 cells for coimmunoprecipitation (co-IP). The results showed that the PRRSV E protein could interact with tetherin. Immunofluorescence microscopy showed that tetherin was primarily located on the surface of uninfected MARC-145 cells and was partially removed from the surface of PRRSV-infected MARC-145 cells (Wang et al., 2014). Generally, virus antagonists can counteract the intrinsic restrictive factors through the following three main mechanisms: (i) coupling the restrictive factors with the protein degradation pathway, (ii) positioning the restrictive factors incorrectly, thus reducing their functional performance, and (iii) use as a mimetic as a limiting factor matrix (Duggal and Emerman, 2012).

In summary, the E protein can interact with the endogenous viral restriction factor tetherin in MARC-145 cells. Due to the lack of a PRRSV E protein antibody and tetherin antibody, the interaction mechanism between tetherin and the E protein on the cell surface could not be detected by flow cytometry. Therefore, based on the interaction mechanism between HIV-2 Env and tetherin (Le Tortorec and Neil, 2009; Hauser et al., 2010), it is speculated that the E protein counteracts the antiviral activity of tetherin by removing its action site from the cell surface, thus preventing recognition by the host’s immune system and regulating the cellular immune response.

## 7. Additional biological functions of the E protein

Interleukin-1 (IL-1) is a highly active proinflammatory cytokine that can lower the pain threshold and damage tissues, causing local inflammation and systemic inflammation. IL-1 has two genotypes, namely, *IL1A* and *IL1B*; the former encodes IL-1α, and the latter encodes IL-1β. IL-1 does not exist in the cells of healthy individuals but is found in a limited number of cell products, such as tissue macrophages, blood monocytes and dendritic cells. It can induce autoinflammation and an innate immune response (Dinarello et al., 2012; Lawson et al., 2012). The precursor of IL-1β is inactive, requiring the activation of inflammatory bodies, causing the activation of caspase-1. The inactive cytoplasmic precursor must be cleaved by caspase-1 to generate the mature active form of IL-1β (Agostini et al., 2004). Porcine alveolar macrophages (PAMs) were pretreated with lipopolysaccharide (LPS) and infected with PRRSV at different multiplicities of infection (MOIs). The concentration of IL-1β in the cell culture was measured by ELISA, and caspase-1 in PAMs was detected by western blotting 72 h after infection. The results showed that the concentration of IL-1β in LPS-primed PAMs increased with increasing MOI and prolonged infection duration, which confirmed that PRRSV could activate the inflammatory corpuscle signal and nuclear factor κB (NF-κB) in PAMs. The inflammatory corpuscle signal is used for the synthesis of pro-IL-1β, and NF-κB is used for the maturation and release of IL-1β. The PRRSV E protein is an ion channel-like protein that can be blocked by amantadine. Therefore, amantadine was used to treat LPS-primed PAMs, which were then infected with PRRSV and transfected with protein E mRNA, after which the release of IL-1β was measured. Among them, transfection of PAMs with protein E mRNA was performed using TransMessenger transfection reagent to transfect PAMs with His-tag-fused protein E mRNA. The results showed that the levels of both PRRSV-infected LPS-primed PAMs and protein E mRNA-transfected amantadine-treated PAMs were significantly decreased in a dose-dependent manner, which indicated that the activity of the E protein channel was related to the activation of inflammatory bodies induced by PRRSV (Zhang et al., 2013).

In summary, the PRRSV E protein is an ion channel protein that can activate inflammatory corpuscle signaling and NF-κB in PAMs by causing disordered ion homeostasis. The former causes the maturation and release of IL-1β, and the latter synthesizes pro-IL-1β, thus inducing tissue inflammation and injury.

## 8. The E protein interacts with host proteins

Cholesterol 25-hydroxylase (CH25H) and 25-hydroxycholesterol (25HC) are hydroxysterols that regulate lipid metabolism. They have many functions in regulating cholesterol homeostasis, inflammation and immune responses and have extensive antiviral activities (Song et al., 2017; Zhao et al., 2020). CH25H is a multitransmembrane endoplasmic reticulum-related enzyme that can catalyze the production of 25HC. The PRRSV E protein can interact with CH25H through the ubiquitin–proteasome pathway, degrade porcine CH25H and inhibit the production of 25HC, thus inhibiting the anti-PRRSV effect of CH25H, promoting the replication of PRRSV and enhancing the inflammatory response mediated by the E protein (Ke et al., 2017, 2019).

Gal-1 is an endogenous innate immune protein in cells that participates in antiviral defense in various ways. Overexpression of Gal-1 inhibits replication of PRRSV. The C-terminal domain (48–73 aa) of the PRRSV E protein interacts with the endogenous innate immunity protein Gal-1, reducing Gal-1 production and promoting PRRSV replication (Li et al., 2019).

HMGB1 is a histone chromatin-binding protein and a proinflammatory cytokine that can enhance the inflammatory response. Protein kinase C (PKC) belongs to a family of serine or threonine kinases that participate in the migration and secretion of HMGB1 in cancer cells or activated monocytes. According to the structural and cofactor requirements, PKC can be divided into three subfamilies: calcium and diacylglycerol-dependent PKCs (cPKCs; α, β and γ), calcium-dependent and DAG-dependent PKCs (nPKCs; δ, ε, η and θ) and calcium and DAG atypical PKCs (aPKCs; ζ, ι and λ). Among these subfamilies, PRRSV E and ORF5a can interact with PKCδ, activate PKC δ secretion, induce HMGB1 secretion, and promote the inflammatory response of PRRSV (Wang et al., 2018).

The E protein of PRRSV is partially located in the mitochondria and can interact with the mitochondrial proteins of host cells (mainly ADP/ATP translocase 3, ATP synthase subunit α (ATP5A), and Prohibitin 2 (PHB)). Upon destruction of the permeability of the mitochondrial membrane, ATP production is affected, and caspase-3 is activated to trigger cell apoptosis during the late stage of PRRSV infection. Immunoprecipitation tandem mass spectrometry (IP-MS) was used to introduce the plasmids pE-EYFP and pEYFP-N1 into HEK 293 cells. The expression of the recombinant E-EYFP protein and EYFP was confirmed by western blotting and by detecting the fluorescence of the transfected cells under a UV microscope. The results showed that 80 cell proteins interacted with the E protein, 25% of which originated from the mitochondria. With a transient expression system and co-IP test, genes encoding flag-tagged porcine mitochondrial proteins were cloned into plasmid vectors, and the DNA of these plasmids was transfected into HEK 293 cells together with the pE-EYFP and pEYFP-N1 vectors. The transfected cell lysate was subjected to co-IP analysis with anti-Flag affinity beads or GFP-Trap beads. The anti-Flag antibody or anti-GFP antibody was used to probe the immune complex decomposed by SDS–PAGE, and it was confirmed that the E protein interacted with three types of mitochondrial proteins (ADP/ATP translocase 3, ATP synthase subunit α (ATP5A) and Prohibitin 2 (PHB)). MARC-145 cells were transfected with pE-EGFP. After 48 h, they were stained with a nontoxic water-soluble water dye. The short-term dynamic localization of E-EGFP in the mitochondria was observed under a confocal microscope. MARC-145 cells were infected with PRRSV, and ATP production was measured at 6, 12, 24, and 48 h after infection. At 24 and 48 h after infection, the ATP level of PRRSV-infected cells decreased significantly. HA-E-expressing alphavirus replicon particle (VRPS)-infected HEK 293 cells were stained with annexin V labeled with Pacific Blue after 48 h. Flow cytometry analysis confirmed that the expression of the E protein in cells induced apoptosis (Pujhari and Zakhartchouk, 2016).

**Figure 2:**
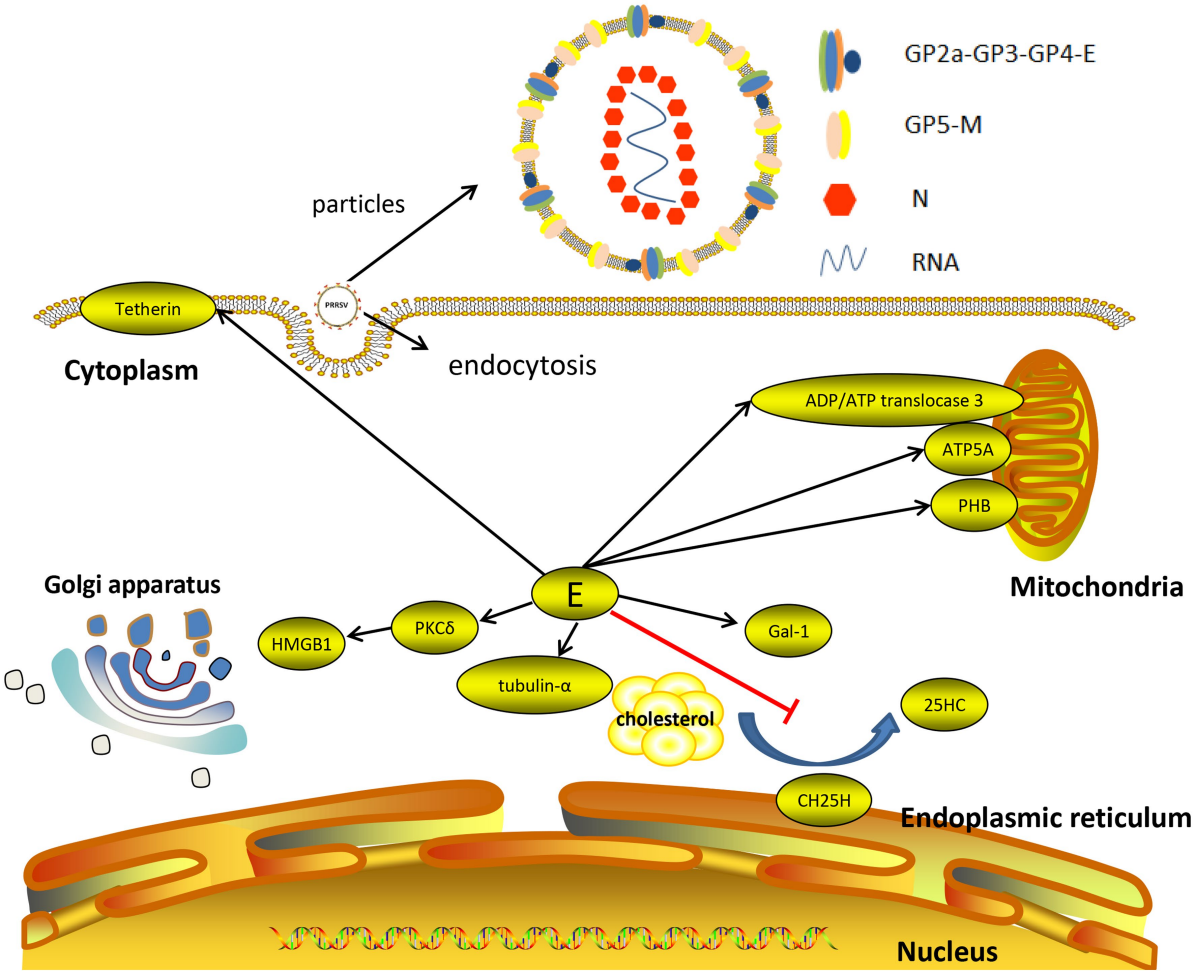
Interaction between the PRRSV E protein and host cells.

In summary, the E protein primarily interacts with seven host proteins, namely, CH25H, Cal-1, PKCδ and mitochondrial proteins (Figure 2). The E protein degrades porcine CH25H and inhibits the production of 25HC through the ubiquitin–proteasome pathway, thus inhibiting the anti-PRRSV effect of CH25H, promoting the replication of PRRSV and enhancing the inflammatory response mediated by the E protein. The E protein can interact with the endogenous natural immune protein Cal-1, reduce the expression of Cal-1 and promote the replication of PRRSV. The E protein and GP5 can interact with PKCδ, which can induce HMGB1 secretion and promote inflammatory reactions by activating PKCδ. The E protein affects ATP production by interacting with mitochondrial proteins and activates capase-3 to trigger cell apoptosis (Pujhari and Zakhartchouk, 2016).

## 9. The E protein interacts with other PRRSV proteins

The GP2a, GP3, and GP4 proteins of PRRSV form a heterotrimer through disulfide bonds, and the E protein forms a heteromultimeric complex with the GP2a-GP3-GP4 heterotrimer through a covalent interaction, and this complex binds to the envelope of virus particles. The E protein and minor structural proteins (GP2a, GP3, and GP4) are strongly interdependent during the binding of virus particles. Following deletion of the GP2a-GP3-GP4 heterotrimer, the E protein content in the virus particles decreased by 60–80%. Upon deletion of the E protein, the binding of the E-GP3-GP4 heterotrimer with virus particles was completely prevented (Wieringa et al., 2004; Chen et al., 2012). A gene knockout experiment showed that deletion of the E protein did not affect the replication of the PRRSV genome and mRNA transcription but affected the infectivity of the virus, which indicated that deletion of the E protein prevented cells infected with PRRSV from producing infectious virus (Wissink et al., 2005). The heteromultimeric complex formed by the PRRSV E protein and GP2a-GP3-GP4 heterotrimer can interact with the CD163 receptor of the host cell through covalent interactions (Das et al., 2010) and mediate the release of the PRRSV genome. CD163 is an indispensable receptor for PRRSV infection *in vivo* and *in vitro* (Burkard et al., 2018). PRRSV first comes in contact with HS on the PAM surface and then switches to a more stable interaction with Sn. After Sn adheres to the virus, the virus-receptor complex is endocytosed via a process mediated by clathrin. After being endocytosed, the virus enters an early inclusion body. Upon acidification by the inclusion body and the action of CD163, the genome of PRRSV is released into the cytoplasm (Chen et al., 2013).

The E protein and N protein form an independent nonvalence connection to cysteine, and the interaction between the N protein and E protein starts after the combination of viral RNA and N protein. The N protein and viral RNA interactions promote the combination of N and E, allowing stable assembly of the core structure of virus particles (Music and Gagnon, 2010).

## 10. E protein and PRRSV infection

The internal structure of the cell consists of the cytoskeleton of the interconnected tubulin network that runs through the cytoplasm. The cytoskeleton consists of actin filaments, intermediate filaments, and microtubules. The cell skeleton plays an important role in cell movement, shape, growth, division, and differentiation and in the movement of intracellular organelles (Fletcher and Mullins, 2010). Cells are connected by nanotubes. PRRSV can make use of the cytoskeletal mechanism of host cells in nanotubes to achieve effective diffusion between cells and transfer viral proteins from one cell to another through the cytoskeleton and nanotubes. This form of virus transport is resistant to the humoral immune response of the host (Guo et al., 2016). The gene encoding Flag-tagged porcine tubulin-α was cloned into the plasmid vector, and the plasmid DNA was transfected into HEK 293 cells together with the pE-EYFP vector. The whole-cell lysate was immunoprecipitated with GFP-Trap and subjected to co-IP. The results confirmed that the PRRSV E protein interacted with porcine tubulin-α. The DNA sequences encoding the N-terminus and C-terminus of the ORF2b gene were cloned into the pEYFP-N1 vector and introduced into the plasmids pN-E-EYFP and pC-E-EYFP, respectively. These plasmids were transfected into HEK 293 cells together with the plasmid for expressing porcine tubulin-α. The transfected cell lysates were subjected to co-IP treatment with Flag and GFP-Trap. The results showed that pC-E-EYFP interacted with porcine tubulin-α, confirming that the C-terminal domain of the E protein was necessary for its interaction with tubulin-α. The use of colchicine, a drug that blocks microtubule polymerization, confirmed that early infection of MARC-145 cells by PRRSV requires microtubule polymerization, but infection at the later stage does not require as extensive a microtubule network (Zhang and Zakhartchouk, 2017).

In summary, the microtubule network is needed to promote virus replication and infection during the prophase of PRRSV infection in MARC-145 cells. The C-terminal domain of residue 25 of the E protein can interact with tubulin-α to promote microtubule depolymerization. Changes in the stability and function of microtubules may lead to apoptosis, thus promoting PRRSV infection.

## 11. E protein and pathogenicity

Yu et al. successively passaged the HP-PRRSV strain JXA1 in MARC-145 cells and performed genomic analyses on P5 and the high-transmission generation (P100-170) JXA1. The results showed that during the excessive attenuation period of JXA1, the virulence determinant of JXA1 was present in both nonstructural and structural proteins, supporting the view that the virulence determinant of PRRSV is multigenic (Yu et al., 2013). Wei et al. used the HBR strain for continuous passage, which also confirmed that the virus attenuation of PRRSV was determined by many structural and nonstructural protein factors (Wei et al., 2013). Chen et al. successively passaged the HP-PRRSV XH-GD strain 122 times in MARC-145 cells and selected 13 different generations of virus for sequencing. The results showed that 50% of 36 amino acid mutations occurred in structural proteins and 50% in nonstructural proteins, among which there were two mutations in the E protein sequence. As an ion channel-like protein, the E protein is embedded in the virus envelope and can form heteropolymers with the GP2a-GP3-GP4 heterotrimers, playing an important role in the virus shelling and genome into cells. Compared with other viruses, the six attenuated viruses have similar mutations at the ninth position of E, with most of them exhibiting D9N, suggesting that E is involved in the attenuation of virulence in the HP-PRRSV XH-GD strain after continuous passage (Chen et al., 2016).

Jiang et al. selected 9 series of Chinese PRRSV passaged strains from GenBank, namely, HuN4, NT0801, JXA1, JX143, GD, GDQY1, BJ, TP and BB0907, for genome analysis (Jiang et al., 2021). The results showed that the amino acid changes in NSP4, NSP9, GP2, E, GP3 and GP4 were consistent during PRRSV attenuation. Among them, there were five amino acid mutations in the E protein during the attenuation of the PRRSV genome, at positions 3, 9, 40, 48, and 62, and the mutation frequency at amino acid position 9 was the highest. It was further confirmed that the E protein is involved in the change in PRRSV virulence, but its degree of participation needs to be further studied (Jiang et al., 2021).

## 12. Drugs affecting the E protein

Amantadine is a proton channel blocker and a commonly used antiviral drug (Wang et al., 1993). Lee et al. treated PRRSV-infected MARC-145 cells with ammonium chloride, chloroquine, amantadine and verapamil. The levels of positive-sense genomic RNA at 2 d post-infection were decreased. The results showed that all four drugs could inhibit the replication of PRRSV, among which ion channel blockers could inhibit the promoter of virus synthesis and greatly reduce the growth rate of PRRSV (Lee and Yoo, 2006).

In summary, as an ion channel-like protein, the E protein can be blocked by amantadine, thus blocking the shelling of PRRSV and affecting PRRSV replication.

## 13. Summary

The PRRSV E protein is a nonglycosylated structural protein with a size of 70–73 aa encoded by the ORF2b gene. The E protein is embedded in the envelope of the virus, has ion channel activity, and can interact with the viral proteins GP2a, GP3 and GP4 to form heterogeneous polymer complexes. These heterogeneous polymer complexes promote the shelling of PRRSV and interact with the host proteins CH25H, Cal-1, PKCδ, and mitochondrial proteins to promote the release of the PRRSV genome and regulate the cellular immune response. As a secondary structural protein, the E protein is not necessary for virus particle assembly. However, in the absence of the E protein, the GP2a-GP3-GP4-E heterogeneous polymer complexes cannot be formed, and the ion channel activity is eliminated, which affects virus shelling. Consequently, the genome cannot be released, and the initiation of viral RNA synthesis is inhibited, which affects the production of PRRSV, and the viral particles lose their infectivity. This characteristic provides new approaches for the design of PRRSV vaccines. At present, there are no specific drugs for PRRSV, and the main reason is that there is not a sufficient understanding of the infection mechanisms of this virus. Therefore, in-depth study of the structural and nonstructural proteins of PRRSV is necessary. This review of the PRRSV E protein provides a theoretical basis for gaining an in-depth understanding of PRRS.

## Author contributions

XC and JP collected data and wrote the original draft. MZ and LH made the final revision of the manuscript. All authors contributed to the article and approved the submitted version.

## Funding

This work received funds from the National Natural Science Foundation of China (31902279). The funder had no role in the study design and collection, analysis, and interpretation of the results.

## Conflict of interest

The authors declare that the research was conducted in the absence of any commercial or financial relationships that could be construed as a potential conflict of interest.

## Publisher’s note

All claims expressed in this article are solely those of the authors and do not necessarily represent those of their affiliated organizations, or those of the publisher, the editors and the reviewers. Any product that may be evaluated in this article, or claim that may be made by its manufacturer, is not guaranteed or endorsed by the publisher.

## References

[ref1] Adams M. J. Lefkowitz E. J. King A. M. Q. Harrach B. Harrison R. L. Knowles N. J.. (2016). Ratification vote on taxonomic proposals to the international committee on taxonomy of viruses (2016). Arch. Virol. 161, 2921–2949. doi: 10.1007/s00705-016-2977-6, PMID: 27424026 PMC7086986

[ref2] Adams M. J. Lefkowitz E. J. King A. M. Q. Harrach B. Harrison R. L. Knowles N. J.. (2017). Changes to taxonomy and the international code of virus classification and nomenclature ratified by the international committee on taxonomy of viruses (2017). Arch. Virol. 162, 2505–2538. doi: 10.1007/s00705-017-3358-5, PMID: 28434098

[ref3] Agostini L. Martinon F. Burns K. McDermott M. F. Hawkins P. N. Tschopp J. (2004). NALP3 forms an IL-1beta-processing inflammasome with increased activity in muckle-Wells autoinflammatory disorder. Immunity 20, 319–325. doi: 10.1016/s1074-7613(04)00046-9, PMID: 15030775

[ref4] Burkard C. Opriessnig T. Mileham A. J. Stadejek T. Ait-Ali T. Lillico S. G.. (2018). Pigs lacking the scavenger receptor cysteine-rich domain 5 of CD163 are resistant to porcine reproductive and respiratory syndrome virus 1 infection. J. Virol. 92, e00415–e00418. doi: 10.1128/JVI.00415-18, PMID: 29925651 PMC6069206

[ref5] Chen Y. He S. Sun L. Luo Y. Sun Y. Xie J.. (2016). Genetic variation, pathogenicity, and immunogenicity of highly pathogenic porcine reproductive and respiratory syndrome virus strain XH-GD at different passage levels. Arch. Virol. 161, 77–86. doi: 10.1007/s00705-015-2597-6, PMID: 26483282

[ref6] Chen J. Liu X. Wang Y. Li L. Hu X. Li N. (2013). Research progress on pathogenic mechanism and disease-resistant breeding of porcine reproductive and respiratory syndrome virus. Anim. Husb. Vet. Med. 44, 1693–1699.

[ref7] Chen H. Zhong F. Li X. Zhang K. Li W. Wen J. (2012). Expression of porcine reproductive and respiratory syndrome virus E protein in porcine alveolar macrophages mediated by lentiviral vector. Anim. Husb. Vet. Med. 44, 254–255.

[ref8] Das P. B. Dinh P. X. Ansari I. H. de Lima M. Osorio F. A. Pattnaik A. K. (2010). The minor envelope glycoproteins GP2a and GP4 of porcine reproductive and respiratory syndrome virus interact with the receptor CD163. J. Virol. 84, 1731–1740. doi: 10.1128/JVI.01774-09, PMID: 19939927 PMC2812361

[ref9] Dinarello C. A. Simon A. van der Meer J. W. M. (2012). Treating inflammation by blocking interleukin-1 in a broad spectrum of diseases. Nat. Rev. Drug Discov. 11, 633–652. doi: 10.1038/nrd3800, PMID: 22850787 PMC3644509

[ref10] Du Y. Zuckermann F. A. Yoo D. (2010). Myristoylation of the small envelope protein of porcine reproductive and respiratory syndrome virus is non-essential for virus infectivity but promotes its growth. Virus Res. 147, 294–299. doi: 10.1016/j.virusres.2009.11.016, PMID: 19951726 PMC7114369

[ref11] Duggal N. K. Emerman M. (2012). Evolutionary conflicts between viruses and restriction factors shape immunity. Nat. Rev. Immunol. 12, 687–695. doi: 10.1038/nri3295, PMID: 22976433 PMC3690816

[ref12] Fletcher D. A. Mullins R. D. (2010). Cell mechanics and the cytoskeleton. Nature 463, 485–492. doi: 10.1038/nature08908, PMID: 20110992 PMC2851742

[ref13] García Durán M. Costa S. Sarraseca J. de la Roja N. García J. García I.. (2016). Generation of porcine reproductive and respiratory syndrome (PRRS) virus-like-particles (VLPs) with different protein composition. J. Virol. Methods 236, 77–86. doi: 10.1016/j.jviromet.2016.03.021, PMID: 27435337

[ref14] Guo B. Chen Z. Liu W. Cui Y. (1996). Study on isolation of PRRSV from fetus suspected of PRRS abortion. Chin. J. Prev. Vet. Med. 18, 3–7.

[ref15] Guo R. Katz B. B. Tomich J. M. Gallagher T. Fang Y. (2016). Porcine reproductive and respiratory syndrome virus utilizes nanotubes for intercellular spread. J. Virol. 90, 5163–5175. doi: 10.1128/JVI.00036-16, PMID: 26984724 PMC4859731

[ref16] Hauser H. Lopez L. A. Yang S. J. Oldenburg J. E. Exline C. M. Guatelli J. C.. (2010). HIV-1 Vpu and HIV-2 Env counteract BST-2/tetherin by sequestration in a perinuclear compartment. Retrovirology 7:51. doi: 10.1186/1742-4690-7-51, PMID: 20529266 PMC2890665

[ref17] Jiang Y. Tong W. Yu L. Li L. Gao F. Li G.. (2021). Identification of virulence associated region during highly pathogenic porcine reproductive and respiratory syndrome virus during attenuation in vitro: complex question with different strain backgrounds. Viruses 14:40. doi: 10.3390/v14010040, PMID: 35062244 PMC8780124

[ref18] Kappes M. A. Faaberg K. S. (2015). PRRSV structure, replication and recombination: origin of phenotype and genotype diversity. Virology 479-480, 475–486. doi: 10.1016/j.virol.2015.02.012, PMID: 25759097 PMC7111637

[ref19] Ke W. Fang L. Jing H. Tao R. Wang T. Li Y.. (2017). Cholesterol 25-hydroxylase inhibits porcine reproductive and respiratory syndrome virus replication through enzyme activity-dependent and -independent mechanisms. J. Virol. 91, e00827–e00817. doi: 10.1128/JVI.00827-17, PMID: 28724759 PMC5599739

[ref20] Ke W. Fang L. Tao R. Li Y. Jing H. Wang D.. (2019). Porcine reproductive and respiratory syndrome virus E protein degrades porcine cholesterol 25-hydroxylase via the ubiquitin-proteasome pathway. J. Virol. 93, e00767–e00719. doi: 10.1128/JVI.00767-19, PMID: 31341055 PMC6798101

[ref21] Keffaber K. (1989). Reproductive failure of unknown etiology. AASP Newsl 1, 1–9.

[ref22] Kreutz L. C. Ackermann M. R. (1996). Porcine reproductive and respiratory syndrome virus enters cells through a low pH-dependent endocytic pathway. Virus Res. 42, 137–147. doi: 10.1016/0168-1702(96)01313-5, PMID: 8806181

[ref23] Lawson S. R. Li Y. Patton J. B. Langenhorst R. J. Sun Z. Jiang Z.. (2012). Interleukin-1β expression by a recombinant porcine reproductive and respiratory syndrome virus. Virus Res. 163, 461–468. doi: 10.1016/j.virusres.2011.11.007, PMID: 22119401 PMC7114469

[ref24] Le Tortorec A. Neil S. J. D. (2009). Antagonism to and intracellular sequestration of human tetherin by the human immunodeficiency virus type 2 envelope glycoprotein. J. Virol. 83, 11966–11978. doi: 10.1128/JVI.01515-09, PMID: 19740980 PMC2772693

[ref25] Lee C. Yoo D. (2005). Cysteine residues of the porcine reproductive and respiratory syndrome virus small envelope protein are non-essential for virus infectivity. J. Gene. Virol. 86, 3091–3096. doi: 10.1099/vir.0.81160-0, PMID: 16227232

[ref26] Lee C. Yoo D. (2006). The small envelope protein of porcine reproductive and respiratory syndrome virus possesses ion channel protein-like properties. Virology 355, 30–43. doi: 10.1016/j.virol.2006.07.013, PMID: 16904148 PMC7111972

[ref27] Li X. Dong Y. Yu H. (2018). Diagnosis, prevention and control of porcine reproductive and respiratory syndrome. Anim. Husb. Vet. 39, 78–79.

[ref28] Li Y. Tas A. Sun Z. Snijder E. J. Fang Y. (2015). Proteolytic processing of the porcine reproductive and respiratory syndrome virus replicase. Virus Res. 202, 48–59. doi: 10.1016/j.virusres.2014.12.027, PMID: 25557977

[ref29] Li Y. Xia Z. Gao Y. Ma (2010). Research progress of structural protein 2b of porcine reproductive and respiratory syndrome virus. Adv. Anim. Med. 31, 99–101. doi: 10.16437/j.cnki.1007-5038.2010.10.020

[ref30] Li L. Zhao K. Gao F. Jiang Y. Shan T. Tong W.. (2019). Restriction of porcine reproductive and respiratory syndrome virus replication by galectin-1. Vet. Microbiol. 235, 310–318. doi: 10.1016/j.vetmic.2019.07.024, PMID: 31383318

[ref31] Liu R. Lin T. Wei Z. Sun L. Lu J. Yuan S. (2011). Research progress of structural proteins of porcine reproductive and respiratory syndrome virus. Adv. Anim. Med. 32, 89–94. doi: 10.16437/j.cnki.1007-5038.2011.12.004

[ref32] Murtaugh M. P. Elam M. R. Kakach L. T. (1995). Comparison of the structural protein coding sequences of the VR-2332 and Lelystad virus strains of the PRRS virus. Arch. Virol. 140, 1451–1460. doi: 10.1007/BF01322671, PMID: 7661696 PMC7086642

[ref33] Music N. Gagnon C. A. (2010). The role of porcine reproductive and respiratory syndrome (PRRS) virus structural and non-structural proteins in virus pathogenesis. Anim. Health Res. Rev. 11, 135–163. doi: 10.1017/S1466252310000034 20388230

[ref34] Opella S. J. (2015). Relating structure and function of viral membrane-spanning Miniproteins. Curr. Opin. Virol. 12, 121–125. doi: 10.1016/j.coviro.2015.05.006, PMID: 26057606 PMC4476644

[ref35] Pinto L. H. Holsinger L. J. Lamb R. A. (1992). Influenza virus M2 protein has ion channel activity. Cells 69, 517–528. doi: 10.1016/0092-8674(92)90452-i 1374685

[ref36] Pujhari S. Zakhartchouk A. N. (2016). Porcine reproductive and respiratory syndrome virus envelope (E) protein interacts with mitochondrial proteins and induces apoptosis. Arch. Virol. 161, 1821–1830. doi: 10.1007/s00705-016-2845-4, PMID: 27068165

[ref37] Ruedas-Torres I. Rodríguez-Gómez I. M. Sánchez-Carvajal J. M. Larenas-Muñoz F. Pallarés F. J. Carrasco L.. (2021). The jigsaw of PRRSV virulence. Vet. Microbiol. 260:109168. doi: 10.1016/j.vetmic.2021.109168, PMID: 34246042

[ref38] Shi M. Lam T. T.-Y. Hon C.-C. Hui R. K.-H. Faaberg K. S. Wennblom T.. (2010). Molecular epidemiology of PRRSV: a phylogenetic perspective. Virus Res. 154, 7–17. doi: 10.1016/j.virusres.2010.08.014, PMID: 20837072

[ref39] Snijder E. J. van Tol H. Pedersen K. W. Raamsman M. J. B. de Vries A. A. F. (1999). Identification of a novel structural protein of Arteriviruses. J. Virol. 73, 6335–6345. doi: 10.1128/JVI.73.8.6335-6345.1999, PMID: 10400725 PMC112712

[ref40] Song Z. Zhang Q. Liu X. Bai J. Zhao Y. Wang X.. (2017). Cholesterol 25-hydroxylase is an interferon-inducible factor that protects against porcine reproductive and respiratory syndrome virus infection. Vet. Microbiol. 210, 153–161. doi: 10.1016/j.vetmic.2017.09.011, PMID: 29103685

[ref41] Sun J. Hao J. Rong F. Jiang L. Jiang L. Bai Y. (2011). Isolation and identification of porcine reproductive and respiratory syndrome virus and sequence analysis of ORF5 gene. China Anim. Husb. Vet. Med. 38, 138–142.

[ref42] Tian Z. Xue J. Huo X. Liu K. Wang X. Yang M.. (2014). Expression of E protein of porcine reproductive and respiratory syndrome virus in Marc-145 cells. Chin. J. Vet. Sci. 34, 546–550. doi: 10.16303/j.cnki.1005-4545.2014.04.005

[ref43] Tian Z. Xue J. Ma D. (2012). Research progress of E protein of porcine reproductive and respiratory syndrome virus. China Anim. Husb. Vet. Med. 39, 42–44.

[ref44] Veit M. Matczuk A. K. Sinhadri B. C. Krause E. Thaa B. (2014). Membrane proteins of arterivirus particles: structure, topology, processing and function. Virus Res. 194, 16–36. doi: 10.1016/j.virusres.2014.09.010, PMID: 25278143 PMC7172906

[ref45] Walker P. J. Siddell S. G. Lefkowitz E. J. Mushegian A. R. Adriaenssens E. M. Alfenas-Zerbini P.. (2021). Changes to virus taxonomy and to the international code of virus classification and nomenclature ratified by the international committee on taxonomy of viruses (2021). Arch. Virol. 166, 2633–2648. doi: 10.1007/s00705-021-05156-1, PMID: 34231026

[ref46] Wang X. Li C. Zhou L. Zhang N. Wang X. Ge X.. (2014). Porcine reproductive and respiratory syndrome virus counteracts the porcine intrinsic virus restriction factors-IFITM1 and Tetherin in MARC-145 cells. Virus Res. 191, 92–100. doi: 10.1016/j.virusres.2014.07.025, PMID: 25102331

[ref47] Wang C. Takeuchi K. Pinto L. H. Lamb R. A. (1993). Ion channel activity of influenza a virus M2 protein: characterization of the amantadine block. J. Virol. 67, 5585–5594. doi: 10.1128/jvi.67.9.5585-5594.1993, PMID: 7688826 PMC237962

[ref48] Wang R. Yang L. Zhang Y. Li J. Xu L. Xiao Y.. (2018). Porcine reproductive and respiratory syndrome virus induces HMGB1 secretion via activating PKC-delta to trigger inflammatory response. Virology 518, 172–183. doi: 10.1016/j.virol.2018.02.021, PMID: 29522984

[ref49] Wei Y. Li S. Huang L. Tang Q. Liu J. Liu D.. (2013). Experimental infection and comparative genomic analysis of a highly pathogenic PRRSV-HBR strain at different passage levels. Vet. Microbiol. 166, 337–346. doi: 10.1016/j.vetmic.2013.05.014, PMID: 23850443

[ref50] Wieringa R. de Vries A. A. F. van der Meulen J. Godeke G.-J. Onderwater J. J. M. van Tol H.. (2004). Structural protein requirements in equine arteritis virus assembly. J. Virol. 78, 13019–13027. doi: 10.1128/JVI.78.23.13019-13027.2004, PMID: 15542653 PMC524988

[ref51] Wissink E. H. J. Kroese M. V. van Wijk H. A. R. Rijsewijk F. A. M. Meulenberg J. J. M. Rottier P. J. M. (2005). Envelope protein requirements for the assembly of infectious Virions of porcine reproductive and respiratory syndrome virus. J. Virol. 79, 12495–12506. doi: 10.1128/JVI.79.19.12495-12506.2005, PMID: 16160177 PMC1211556

[ref52] Wu W. H. Fang Y. Farwell R. Steffen-Bien M. Rowland R. R. Christopher-Hennings J.. (2001). A 10-kDa structural protein of porcine reproductive and respiratory syndrome virus encoded by ORF2b. Virology 287, 183–191. doi: 10.1006/viro.2001.1034, PMID: 11504553

[ref53] Wu W.-H. Fang Y. Rowland R. R. R. Lawson S. R. Christopher-Hennings J. Yoon K.-J.. (2005). The 2b protein as a minor structural component of PRRSV. Virus Res. 114, 177–181. doi: 10.1016/j.virusres.2005.06.014, PMID: 16095746 PMC7127422

[ref54] Xu Y. Hou P. Wang H. Zhao G. Pan W. He H. (2019). The role of Viroporins in virus infection. Adv. Anim. Med. 40, 106–109. doi: 10.16437/j.cnki.1007-5038.2019.08.017

[ref55] Yi X. Wei P. Wang X. (2014). Research progress on antiviral mechanism of natural immune limiting factor Tetherin. Prog. Biochem. Biophys. 41, 32–40.

[ref56] Yu X. Chen N. Deng X. Cao Z. Han W. Hu D.. (2013). Genomic sequencing reveals mutations potentially related to the Overattenuation of a highly pathogenic porcine reproductive and respiratory syndrome virus. Clin. Vaccine Immunol. 20, 613–619. doi: 10.1128/CVI.00672-12, PMID: 23408525 PMC3623411

[ref57] Yu M. Liu X. Sun L. Chen C. Ma G. Kitamura Y.. (2010). Subcellular localization and topology of porcine reproductive and respiratory syndrome virus E protein. Virus Res. 152, 104–114. doi: 10.1016/j.virusres.2010.06.012, PMID: 20600392

[ref58] Yuan S. Tang D. Yang Z. Han C. Yan R. Chen C.. (2022). Research progress on structural protein function of porcine reproductive and respiratory syndrome virus. North Anim. Husb. 8:25.

[ref59] Zhang K. Hou Q. Zhong Z. Li X. Chen H. Li W.. (2013). Porcine reproductive and respiratory syndrome virus activates inflammasomes of porcine alveolar macrophages via its small envelope protein E. Virology 442, 156–162. doi: 10.1016/j.virol.2013.04.007, PMID: 23664331

[ref60] Zhang S. Shen Z. Liu L. Ma Y. Liu J. (2012). Research progress on biological functions of structural and nonstructural proteins of porcine reproductive and respiratory syndrome virus. J. Anim. Husb. Vet. Med. 43, 1683–1696.

[ref61] Zhang M. Zakhartchouk A. (2017). Porcine reproductive and respiratory syndrome virus envelope (E) protein interacts with tubulin. Vet. Microbiol. 211, 51–57. doi: 10.1016/j.vetmic.2017.10.002, PMID: 29102121

[ref62] Zhao J. Chen J. Li M. Chen M. Sun C. (2020). Multifaceted functions of CH25H and 25HC to modulate the lipid metabolism, immune responses, and broadly antiviral activities. Viruses 12:E727. doi: 10.3390/v12070727, PMID: 32640529 PMC7411728

